# Relationship Between Expansion and Strength of Cement Paste with CaO-Based Expansive Agent

**DOI:** 10.3390/ma17246125

**Published:** 2024-12-14

**Authors:** Tengfei Hua, Zhiwei Cao, Shijun Zhang, Rongxin Guo

**Affiliations:** 1 Yunnan Science and Technology Research Institute of Highway, Kunming 650051, China; huatengfei0209@126.com (T.H.); czw770726@163.com (Z.C.); 2 Faculty of Civil Engineering and Mechanics, Kunming University of Science and Technology, Kunming 650500, China

**Keywords:** CaO-based expansive agent, expansion rate, strength, microstructure, hydration products

## Abstract

In order to explore the intrinsic relationship between the expansion and strength of cementitious materials with CaO-based expansion agent (CEA), the effects of CEA on the expansion rate, strength, microstructure, and hydration products of cement paste were studied. The interaction mechanism between the expansion rate and compressive strength of cement paste with CEA was discussed. The results show that the addition of CEA increases the expansion rate, porosity, and hydration degree of cement paste while reducing the compactness and compressive strength of cement paste. As the CEA dosage increases, the expansion rate of cement paste gradually increases. During the expansion of cement paste, microcracks may occur. The larger the expansion rate, the more microcracks there are, and the compressive strength decreases linearly with the increase in the expansion rate. When the CEA dosage is the same, the expansion rate of cement paste gradually increases with the increase of mineral admixture content, and the expansion rate decreases linearly with the increase of compressive strength.

## 1. Introduction

As a large building material for contemporary infrastructure construction, concrete is widely used in construction, water conservancy and hydropower, highway, railway, and municipal engineering. However, with the development of concrete material technology, the material characteristics of increasingly complex concrete composition, the increased fluidity, and accelerated early strength development lead to increased concrete shrinkage. The long-span, large-volume, strong constraint structure, as well as high temperature, dry, and other harsh environments, lead to the prominent problem of concrete shrinkage cracking [1]. If the initial support and secondary lining concrete of highway or railway tunnels cracks, water and corrosive ions in the surrounding rock will quickly invade the interior of the concrete through the cracks, causing the performance deterioration of the tunnel concrete lining and seriously affecting the service performance and vehicle safety of the tunnel [2,3,4]. Therefore, in order to truly improve the durability of concrete structures in practical engineering, it is necessary to fundamentally solve the problem of early shrinkage cracking of concrete [1].

Compensating for concrete shrinkage by using expansive agents is an effective method to suppress concrete shrinkage cracking [5,6,7]. Expansive agents can be divided into CaO-based expansive agent (CEA), calcium sulphoaluminate expansive agent, and MgO-based expansive agent based on their different expansive components. The main expansive component of CEA is f-CaO, which reacts with water to generate Ca(OH)_2_. Ca(OH)_2_ crystallizes in situ around CEA and generates expansion stress under the constraint of the surrounding matrix, causing the volume expansion of cementitious materials and compensating for their shrinkage [8,9,10]. CEA has the characteristics of fast expansion speed, high expansion energy, and low water demand [11,12] and has been applied in the preparation of compensating shrinkage concrete in engineering [1,7].

According to the expansion mechanism of CEA, the expansion stress increases with the increase of CEA dosage, leading to the increase of expansion. Zhao et al. [13] found that when the dosage of CEA was 3% and 6%, the 28 d autogenous shrinkage of concrete decreased by 77.65% and 105.35%, respectively, compared to the reference. Meanwhile, the compressive strength, splitting tensile strength, and elastic modulus of concrete with 3% and 6% CEA decreased at 3 d, 7 d, 14 d, and 28 d. This was due to the coarsened pore size and increased porosity of cementitious materials caused by the addition of CEA [14]. Wang et al. [15] found that when the CEA dosage gradually increased from 0% to 12%, the expansion rate of concrete gradually increased, and the compressive strength first increased and then gradually decreased. Wang et al. [15] believed that an appropriate amount of expansion can improve the compactness of concrete. It can be seen that the mechanical properties of concrete are affected by expansion.

Wang et al. [15] also showed that the expansion rate of concrete with a lower w/b (0.33) was lower than that of concrete with a higher w/b (0.43) when the CEA dosage was the same. This might be because the smaller the w/b, the higher the strength of the concrete and the greater the constraint on the expansion stress, resulting in a decrease in the expansion rate. It was also possible that concrete with a lower w/b had a lower water content, which cannot fully meet the hydration requirements of CEA and led to a decrease in expansion rate. In addition, adding mineral admixtures such as fly ash and slag reduced the expansion rate of concrete [15]. Li et al. [16] suggested that fly ash and slag will consume part of the expansion product Ca(OH)_2_ due to pozzolanic reaction, resulting in a decrease in expansion. However, the addition of mineral admixtures such as fly ash and slag can reduce the early strength of cementitious materials [17]. In theory, the smaller the strength, the greater the expansion. Therefore, the expansion of concrete is affected by strength, and there is no unified understanding of the effect of mineral admixtures on the expansion of cementitious materials.

In summary, the expansion of cementitious materials may affect their mechanical properties, and a change of the strength of cementitious materials can also affect the expansion. However, the relationship between the expansion and the development of mechanical properties in existing research is still unclear, which is not conducive to the application of CEA in different types of cementitious materials. In this paper, pure cement pastes, cement–slag composite pastes, and cement–fly ash composite pastes mixed with CEA were taken as the research objects. The influences of CEA dosage, slag, and fly ash content on the expansion rate, flexural strength, compressive strength, microstructure, and hydration products of cement pastes were studied, and the internal relationship between the expansion and strength of cement pastes with CEA was deeply explored.

## 2. Materials and Methods

### 2.1. Materials and Mixtures

**Figure 1:**
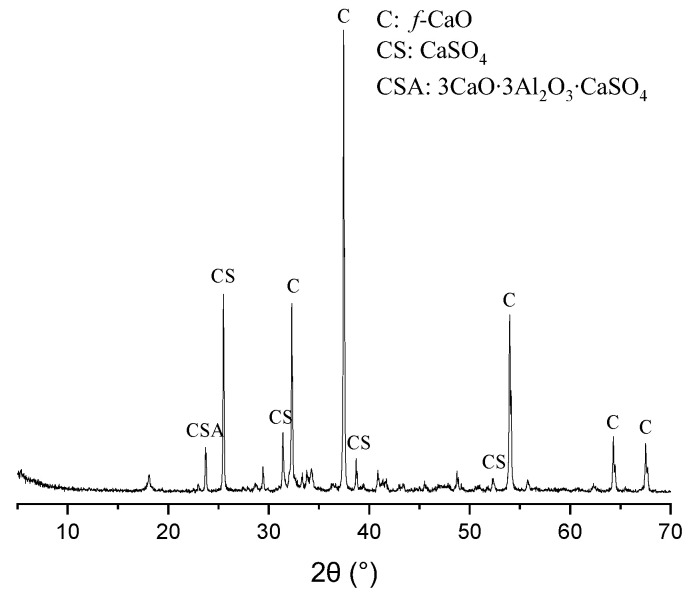
XRD pattern of CEA.

In this study, cement is P·I 42.5 according to Chinese standards [18]. The specific surface areas of cement, slag, fly ash, and CEA are 356, 471, 552, and 292 m^2^/kg, respectively, and the main chemical compositions of cement, slag, fly ash, and CEA are shown in Table 1. The X-ray diffraction (XRD, D8 ADVANCE X-RAY DIFFRACTOMETER, Bruker, Germany) pattern of CEA is shown in Figure 1. The main ingredients of CEA are free lime (f-CaO), anhydrite (CaSO_4_), and calcium sulphoaluminate (3CaO·3Al_2_O_3_·CaSO_4_).

The mix proportions of cement pastes are shown in Table 2. The cementitious materials include cement, slag, fly ash, and CEA. P0.4E4 indicates that cement paste is pure cement paste with w/b of 0.4 and 4% CEA. P0.4S60E4 indicates that cement paste is a cement–slag composite paste with w/b of 0.4, 60% slag, and 4% CEA. P0.4F60E4 indicates that cement paste is a cement–fly ash composite paste with w/b of 0.4, 60% FA, and 4% CEA.

### 2.2. Test Methods

Three 25 mm × 25 mm × 280 mm prisms as one group were formed, which were cured in the standard curing box (20 °C ± 1 °C, RH > 95%) for 24 h and then demoulded. The initial lengths of prisms were tested immediately with a length meter, and then the prisms were immersed in water for curing (20 °C ± 1 °C). After curing in water to the corresponding age, the lengths of prisms were tested again. Finally, according to [19], the expansion rates of the prisms at a certain age were calculated. The average expansion rate of the three prisms is shown in this paper.

Three 40 mm × 40 mm × 160 mm prisms as one group were formed, which were cured in the standard curing box (20 °C ± 1 °C, RH > 95%) for 24 h and then demoulded. The prisms were immersed in water for curing (20 °C ± 1 °C). After curing in water to the corresponding age, the flexural strength and compressive strength of the prisms were tested according to [20]. The average value of the flexural strength and compressive strength of the three prisms is shown in this paper.

Three 40 mm × 40 mm × 160 mm prisms as one group were formed, which were cured in the standard curing box (20 °C ± 1 °C, RH > 95%) for 9 h and then demoulded. The ultrasonic wave velocity of the prisms was detected by NM-4B non-metal ultrasonic tester (Koncrete Engineering Detection Co., Ltd., Beijing, China), and then the prisms were immersed in water for curing (20 °C ± 1 °C). After curing in water to the corresponding age, the ultrasonic wave velocity of the prisms was measured again. The average ultrasonic wave velocity of three prisms is shown in this paper.

The prism cured to a certain age was cut into 5 mm thick sheets, and the sheets were cut with scissors to prepare the paste particles with a particle size of about 3 mm. The paste particles were soaked in anhydrous ethanol for 7 d and then dried at 45 °C in a vacuum drying oven for 2 d. Finally, the pore structure of hardened cement paste was measured by mercury intrusion porosimetry (MIP). The MicroActive AutoPore V 9600 with applied pressures of 0.5 to 62,354.6 psia, corresponding to the pore size of 2.9 nm to 362 μm, was applied in this study.

The cement paste was broken at a certain age, and the core samples were soaked in anhydrous ethanol for 24 h to stop hydration. The core samples were dried in a vacuum oven at 45 °C for 2 d, ground into powder, and passed through 80 μm square pore sieve. The XRD pattern of the powder was tested by A D8 ADVANCE X-RAY DIFFRACTOMETER (Bruker, Germany). In addition, some powder was weighed and placed in a muffle furnace, calcined at 950 °C for 2 h, and weighed to obtain the weight of the calcined powder, and the water content in the powder was calculated, which was the non-evaporable water content in cement paste.

## 3. Results

### 3.1. Expansion Rate

**Figure 2:**
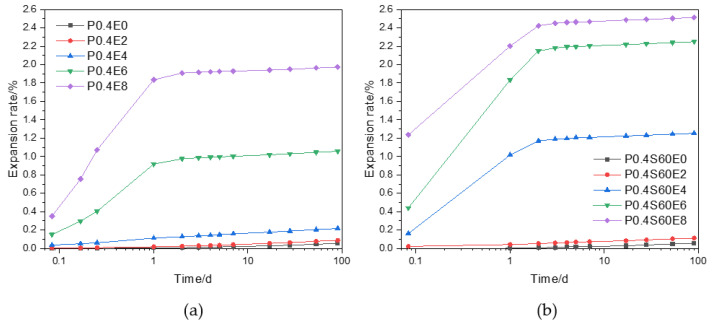
Effect of CEA dosage on the water-curing expansion rate of cement paste: (**a**) pure cement paste; (**b**) cement–slag composite paste.

Figure 2 shows the expansion rate with the age of pure cement paste and cement–slag composite paste with different CEA dosages. It can be seen that under the water curing, the pure cement paste and cement–slag composite paste without CEA showed a phenomenon of moisture expansion, and the expansion rate of cement paste gradually increased with the increase of curing age. This may be due to the gradual increase of water penetrating into cement paste with the increase of curing age, resulting in greater moisture expansion. For pure cement pastes, as shown in Figure 2a, adding CEA increased the expansion rate of cement paste, and the expansion rate increased significantly with the increase of CEA dosage. When the dosage of CEA was 2%, 4%, 6%, and 8%, the 1 d expansion rate of cement paste was 0.017%, 0.111%, 0.918%, and 1.836%, respectively. Compared with cement paste without CEA, the 1 d expansion rate increased by 0.012%, 0.106%, 0.913%, and 1.831%, respectively. In addition, when the CEA dosage was 2% and 4%, the expansion rate of cement paste increased slowly with the increase of curing age from 0.006% and 0.035% at 2 h to 0.087% and 0.216% at 90 d, respectively. However, when the CEA dosage was 6% and 8%, the expansion rate of cement paste increased sharply within 1 d from 0.150% and 0.350% at 2 h to 0.918% and 1.836% at 1 d, respectively. The expansion rate increased slowly after 1 d from 0.918% and 1.836% at 1 d to 1.057% and 1.975% at 90 d, respectively.

For cement–slag composite paste, as shown in Figure 2b, similar to the pure cement paste, the addition of CEA increased the expansion rate of cement paste, and the expansion rate increased significantly with the increase of CEA dosage. When the CEA dosage was 2%, the expansion rate of cement paste increased slowly with the increase of curing age from 0.021% at 2 h to 0.111% at 90 d. Different from the pure cement paste, the expansion rate of cement paste increased sharply within 2 d when the CEA dosage was 4%, 6%, and 8% from 0.161%, 0.440%, and 1.236% at 2 h to 1.171%, 2.150%, and 2.424% at 2 d, respectively. After 2 d, the expansion rate increased slowly from 1.171%, 2.150%, and 2.424% at 2 d to 1.252%, 2.250%, and 2.513% at 90 d, respectively. Compared with Figure 2a and Figure 2b, the expansion rates of cement–slag composite paste were greater than that of pure cement paste with the same CEA dosage.

The more the CEA, the greater the expansion stress, so the expansion rate of cement paste is greater [13]. Previous studies [9,21] have pointed out that the hydration of CEA in cementitious materials is mainly concentrated within 3 d. When the CEA dosage was low, the expansion stress caused by the hydration of CEA was small, which was not enough to make cement paste expand significantly under the constraint of cement paste. When the CEA dosage was high, the high expansion stress produced in the early stage (within 3 d) made cement paste expand significantly. The early strength of cement–slag composite paste was small, and the constraint on the expansion stress was small, leading to a larger expansion.

**Figure 3:**
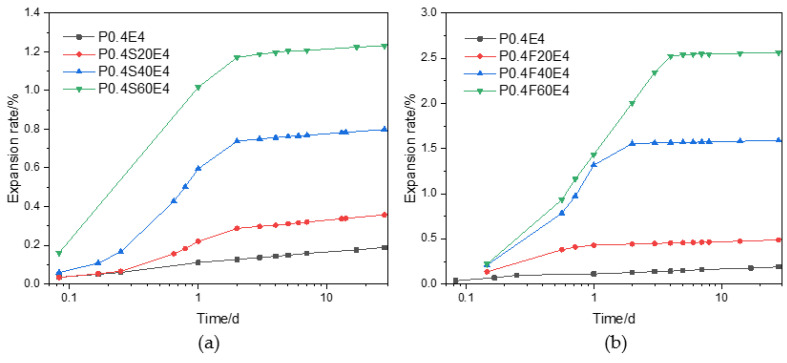
Effect of CEA on the water-curing expansion rate of cement paste with different content of slag and fly ash: (**a**) cement–slag composite paste; (**b**) cement–fly ash composite paste.

Figure 3 plots the expansion rate with the age of cement pastes with different slag and fly ash contents and the same CEA dosage. As shown in Figure 3a, the expansion rate of cement pastes with 4% CEA gradually increased with the increase of slag content at the same curing age. When the slag content was 20%, 40%, and 60%, the expansion rate of cement paste increased sharply within 2 d from 0.034%, 0.059%, and 0.161% at 2 h to 0.288%, 0.738%, and 1.171% at 2 d, respectively. After 2 d, the expansion rate increased slowly from 0.288%, 0.738%, and 1.171% at 2 d to 0.357%, 0.799%, and 1.231% at 28 d, respectively. The addition of slag may reduce the early strength of cement paste, resulting in the decrease of the restraining force on the expansion. Under the same expansion stress, the smaller the restraining force, the larger the expansion rate. Therefore, as the slag content increased, the expansion rate of cement paste increased.

As shown in Figure 3b, similar to the cement–slag composite paste, when the CEA content was 4%, the expansion rate of the cement–fly ash composite paste gradually increased with the increase of the fly ash content at the same curing age. The expansion rate of the cement–fly ash composite paste rapidly increased at an early age and then slowly increased with age. When the fly ash content was 20%, 40%, and 60%, the 2 d expansion rate of cement paste was 0.442%, 1.556%, and 2.004%, which was significantly higher than that of cement paste with the same slag content. Due to the lower activity of fly ash than slag, the strength of cement–fly ash composite paste was less than that of cement–slag composite paste, and the expansion rate of cement–fly ash composite paste with a smaller restraining force was greater than that of cement–slag composite paste under the same expansion stress.

Comprehensive analysis of the results in Figure 2 and Figure 3 demonstrates that the addition of CEA can significantly increase the expansion rate of cement paste. The expansion rate is affected by the CEA dosage and type of cement paste, and the expansion window of cement paste with CEA is mainly within 3 d. The expansion rate of cement paste increases with the increase of CEA dosage and slag (or fly ash) content.

### 3.2. Strength

**Figure 4:**
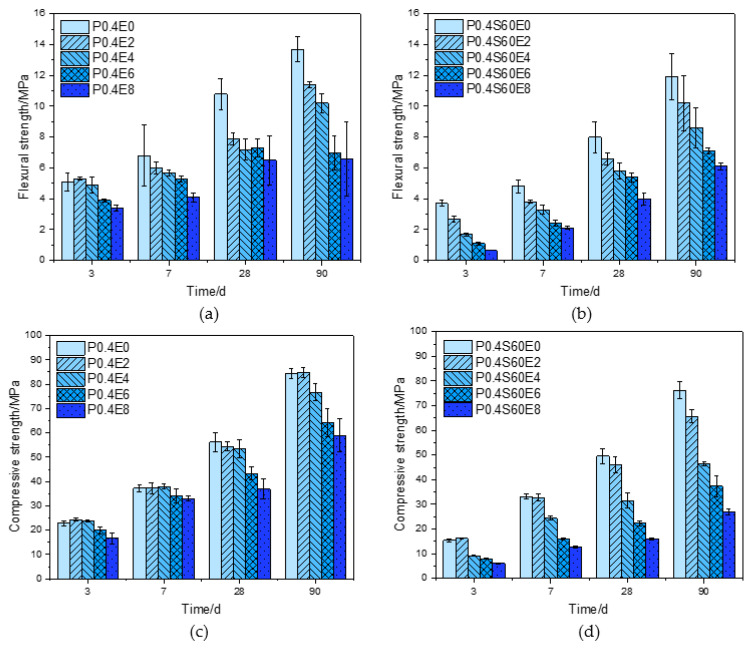
Effect of CEA dosage on the strength of cement paste: (**a**) flexural strength of pure cement paste; (**b**) flexural strength of cement–slag composite paste; (**c**) compressive strength of pure cement paste; (**d**) compressive strength of cement–slag composite paste.

Figure 4 shows the flexural strength and compressive strength of pure cement paste and cement–slag composite paste with different CEA dosages at different ages. For pure cement paste, as shown in Figure 4a, the 3 d flexural strength of cement paste with 2% CEA was slightly greater than that of cement paste without CEA. The 7 d, 28 d, and 90 d flexural strength of cement paste decreased with the increase of CEA dosage. As shown in Figure 4c, when the CEA dosages were 2% and 4%, the 3 d and 7 d compressive strengths of cement pastes were slightly higher than that of cement paste without CEA. The 28 d and 90 d compressive strength of cement paste decreased with the increase of CEA dosage. Compared with cement paste without CEA, the 28 d compressive strengths of cement pastes with 2%, 4%, 6%, and 8% CEA decreased by 3.2%, 5.0%, 23.1%, and 34.2%, respectively.

For cement–slag composite paste, as shown in Figure 4b,d, the flexural strength of cement paste gradually decreased with the increase of CEA dosage. The 3 d compressive strength of cement paste with 2% CEA was slightly higher than that of cement paste without CEA. The 7 d, 28 d, and 90 d compressive strength of cement paste gradually decreased with the increase of CEA dosage. Compared with cement paste without CEA, the 28 d compressive strengths of cement pastes with 2%, 4%, 6%, and 8% CEA decreased by 7.1%, 36.4%, 54.9%, and 67.7%, respectively. In addition, compared with pure cement paste, the strength of cement–slag composite paste with the same CEA dosage was smaller.

**Figure 5:**
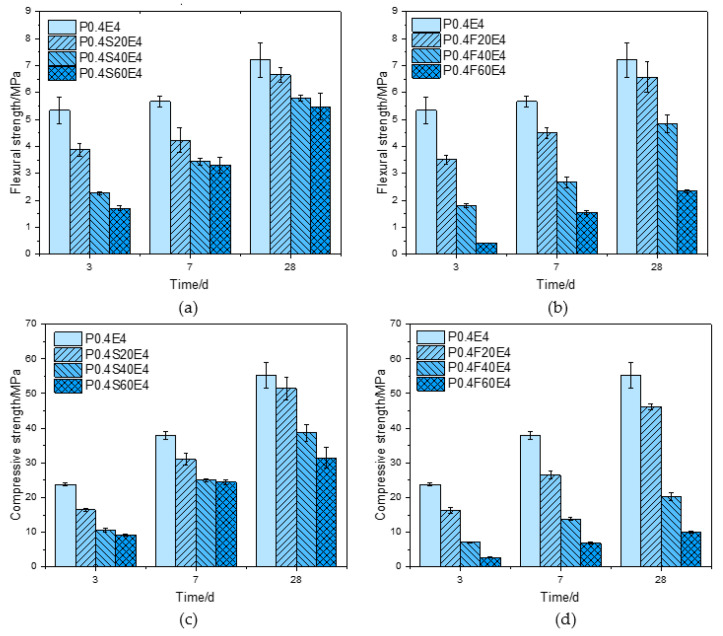
Effect of CEA on the strength of cement paste with different slag and fly ash contents: (**a**) flexural strength of cement–slag composite paste; (**b**) flexural strength of cement–fly ash composite paste; (**c**) compressive strength of cement–slag composite paste; (**d**) compressive strength of cement–fly ash composite paste.

Figure 5 shows the flexural strength and compressive strength of cement pastes with the same CEA dosage and different slag and fly ash contents at different ages. As shown in Figure 5a,c, the flexural strength and compressive strength of cement paste with 4% CEA at the same curing age gradually decreased with the increase of slag content. Compared with pure cement paste, the 3 d compressive strength of cement paste with 20%, 40%, and 60% slag decreased by 30.8%, 55.4%, and 61.3%, respectively, and the 28 d compressive strength decreased by 6.8%, 30.0%, and 43.0%, respectively. It was observed that the compressive strength loss at an early age decreased at a later age.

As shown in Figure 5b,d, similar to the cement–slag composite paste, when the CEA content was 4%, the flexural strength and compressive strength of cement paste with 4% CEA gradually decreased with the increase of the fly ash content at the same curing age. Compared with pure cement paste, the 3 d compressive strength of cement paste with 20%, 40%, and 60% fly ash decreased by 31.4%, 70.2%, and 88.4%, respectively, and the 28 d compressive strength decreased by 16.4%, 63.4%, and 81.7%, respectively. Obviously, the strength loss of cement–fly ash composite paste caused by the same expansion stress was greater than that of cement–slag composite paste.

In summary, the early strength of cement paste is increased by adding a lower dosage of CEA, but the strength of cement paste at each age gradually decreases with the increase of CEA dosage. When the dosage of CEA is the same, the strength reductions of different types of cement pastes from large to small are cement–fly ash composite paste, cement–slag composite pastes, and pure cement paste, respectively. When the CEA dosage is low, the expansion stress is small, and part of the expansion product Ca(OH)_2_ may fill the pores of the early cement paste, resulting in the increase of the compactness of cement paste, thus increasing the early strength of cement paste. However, with the increase of CEA dosage, the expansion stress gradually increases, which may cause microcracks in cement paste, thus reducing the strength of cement paste [22]. Under the same expansion stress, the cement–fly ash composite paste produces the most microcracks internally, followed by the cement–slag composite paste, and the pure cement paste produces the least microcracks internally, resulting in a decrease of strength reduction in turn.

### 3.3. Microstructure

**Figure 6:**
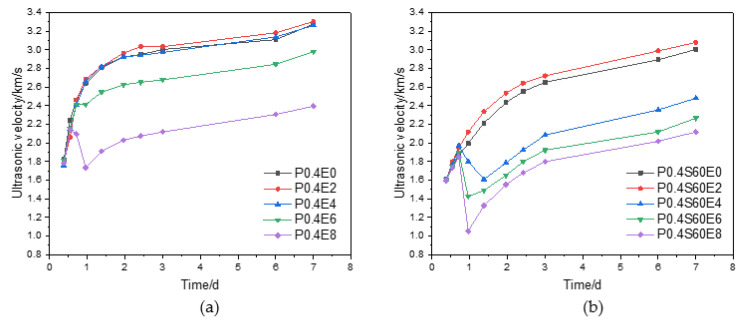
Effect of CEA dosage on compactness of cement paste: (**a**) pure cement paste; (**b**) cement–slag composite paste.

According to the results of the expansion rate and strength of cement paste, the expansion window period of CEA in cement paste is within 3 d and may lead to the strength reduction of cement paste. The ultrasonic velocity through cement paste can be used to characterize the compactness, and the ultrasonic velocity is positively correlated with the compactness of cement paste [23]. Figure 6 plots the ultrasonic velocity with age through cement paste with different dosages of CEA. For pure cement paste, as shown in Figure 6a, when the dosage of CEA was 0%, 2%, and 4%, the ultrasonic velocity of cement paste gradually increased with the increase of age, and the ultrasonic velocities of cement pastes with 2% and 4% CEA were slightly higher than that of cement paste without CEA. This is consistent with the results that the 3 d and 7 d compressive strengths of cement pastes with 2% and 4% CEA were slightly higher than that of cement paste without CEA, indicating there was a good correlation between ultrasonic velocity and compressive strength. When the dosages of CEA were 6% and 8%, the ultrasonic velocity of cement paste stopped increasing or even decreased sharply at a certain age and then gradually increased with age, but the ultrasonic velocities were significantly lower than that of cement paste without CEA. The ultrasonic velocity of cement paste with 6% CEA was 2.42 km/s at 17 h, and it almost did not increase at 23 h and then gradually increased. The 3 d and 7 d ultrasonic velocities of cement paste were only 2.68 km/s and 2.98 km/s, which were significantly lower than 3 km/s and 3.28 km/s of cement paste without CEA. The ultrasonic velocity of cement paste with 8% CEA decreased sharply from 2.14 km/s at 13 h to 1.73 km/s at 23 h and then increased gradually. The 3 d and 7 d ultrasonic velocities of cement paste were only 2.12 km/s and 2.40 km/s, which were smaller than that of cement paste with 6% CEA.

For cement paste without CEA, due to the continuous hydration of cement, the compactness of cement paste gradually increased with age, so the ultrasonic velocity through cement paste gradually increased. For cement paste with a large dosage of CEA, the compactness of cement paste increased with age before the rapid hydration of CEA, so the ultrasonic velocity through cement paste specimen increased gradually. Subsequently, the rapid hydration of CEA produced expansion stress. When the expansion stress was greater than the tensile strength of cement paste, microcracks were generated, which significantly reduced the compactness of cement paste, resulting in a significant reduction in the ultrasonic velocity through cement paste specimen. Due to the continuous hydration of cement, the compactness of cement paste gradually increased with age, so the ultrasonic velocity through the cement paste specimen gradually increased.

For the cement–slag composite paste, as shown in Figure 6b, the ultrasonic velocity of cement paste with 2% CEA was slightly higher than that of cement paste without CEA, which corresponds to that of the 3 d compressive strength of cement paste, which was slightly higher than that of cement paste without CEA. When the dosages of CEA were 4%, 6%, and 8%, the ultrasonic velocity of cement paste decreased sharply at a certain age and then increased gradually with age, and the ultrasonic velocities were significantly lower than that of cement paste without CEA. The ultrasonic velocity of cement paste with 4% CEA decreased sharply from 1.97 km/s at 17 h to 1.61 km/s at 33 h. The ultrasonic velocity of cement paste with 6% CEA decreased sharply from 1.90 km/s at 17 h to 1.43 km/s at 23 h. The ultrasonic velocity of cement paste with 8% CEA decreased sharply from 1.85 km/s at 17 h to 1.05 km/s at 23 h. It can be concluded that the larger the CEA dosage, the smaller the ultrasonic velocity and the greater the reduction of ultrasonic velocity. The time period of rapid reduction of ultrasonic velocity is also in the rapid expansion period of cement paste. Compared with pure cement paste, the ultrasonic velocity of cement–slag composite paste is smaller, which is consistent with the result of compressive strength.

**Figure 7:**
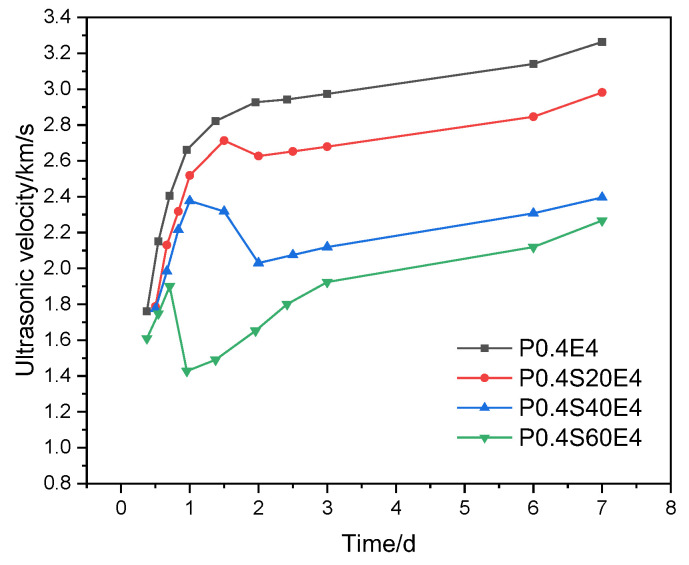
Effect of CEA on compactness of cement paste with different slag contents.

Figure 7 plots the ultrasonic velocity with age through cement paste with 4% CEA and different slag contents. When the CEA dosage was 4%, the ultrasonic velocity of cement paste decreased with the increase of slag content at the same age, which is consistent with the strength results in Figure 5. When the slag contents were 20%, 40%, and 60%, the ultrasonic velocities of cement pastes decreased sharply at a certain age and then increased gradually with age. The ultrasonic velocity of cement paste with 20% slag decreased from 2.71 km/s at 33 h to 2.63 km/s at 47 h. The ultrasonic velocity of cement paste with 40% slag decreased from 2.38 km/s at 23 h to 2.03 km/s at 47 h. The ultrasonic velocity of cement paste with 60% slag decreased from 1.90 km/s at 17 h to 1.43 km/s at 23 h. From this, with the increase of slag content, the age at which the ultrasonic velocity began to decrease gradually advanced, and the reduction of ultrasonic velocity gradually increased. This means that when the CEA dosage is the same, the smaller the strength of cement paste, the earlier the time leading to the reduction of compactness and the greater the reduction of compactness.

In general, when the dosage of CEA is small, the expansion rate of cement paste gradually increases with age, and there is no rapid expansion stage. The compactness of cement paste at an early age does not decrease or is even slightly greater than that of cement paste without CEA, so the early strength of cement paste increases slightly. When the dosage of CEA is large, cement paste expands rapidly in the early age, and the time period when the compactness of cement paste decreases significantly lies in the rapid expansion window of cement paste, which leads to the reduction of the strength of cement paste.

**Figure 8:**
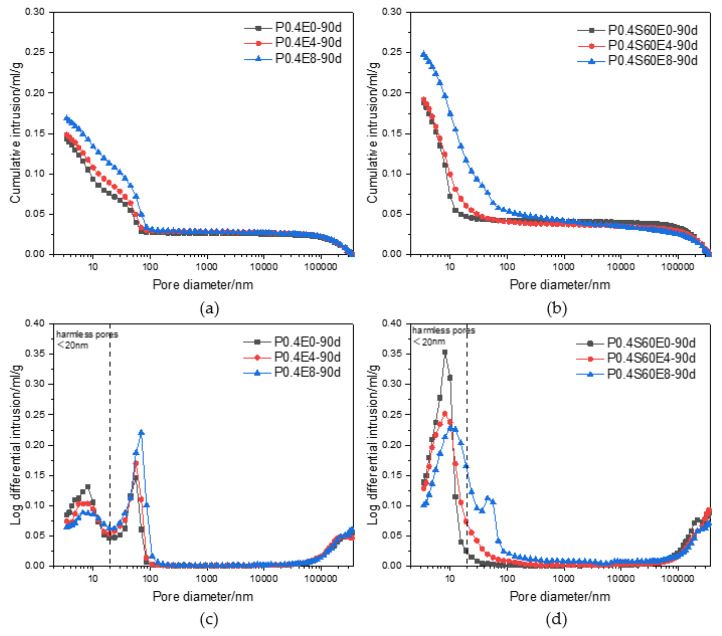
Effect of CEA on pore size distribution of cement paste: (**a**) cumulative pore volume of pure cement paste; (**b**) cumulative pore volume of cement–slag composite paste; (**c**) increment pore volume of pure cement paste; (**d**) increment pore volume of cement–slag composite paste.

In order to explore the effect of CEA on the microstructure of long-age cement paste, the pore structure characteristics of cement pastes with different CEA dosages at 90 d were tested by MIP. Wu [24] classified the pores of concrete according to the degree of influence on the compressive strength as harmless pores (<20 nm), less harmful pores (20 nm–50 nm), harmful pores (50 nm–200 nm), multiple harmful pores (>200 nm). Figure 8 shows the pore size distributions of cement pastes with different CEA dosages at 90 d. For pure cement paste, as shown in Figure 8a, the cumulative pore volume of cement paste gradually increased with the increase of CEA dosage. This shows that the addition of CEA increases the pore content of cement paste, and the pore content gradually increases with the increase of CEA dosage. As shown in Figure 8c, as the CEA dosage increased, the increment of harmless pores volume in cement paste gradually decreased, and the increment of less harmful pores and harmful pores volume gradually increased. This means that adding CEA may coarsen the pore size of cement paste and increase the content of less harmful and harmful pores.

For cement–slag composite paste, as shown in Figure 8b,d, similar to pure cement paste, with the increase of CEA dosage, the cumulative pore volume of cement paste gradually increased, the increment of harmless pores volume in cement paste gradually decreased, and the increment of less harmful pores and harmful pores volume gradually increased. The cumulative pore volume of cement–slag composite paste was larger than that of pure cement paste with the same CEA dosage. In addition, with the increase of CEA dosage, the reduction of harmless pores volume and the increase of less harmful pores and harmful pores volume were significantly greater than those of pure cement paste. This means that CEA has a greater influence on the pore structure of cement–slag composite paste.

**Figure 9:**
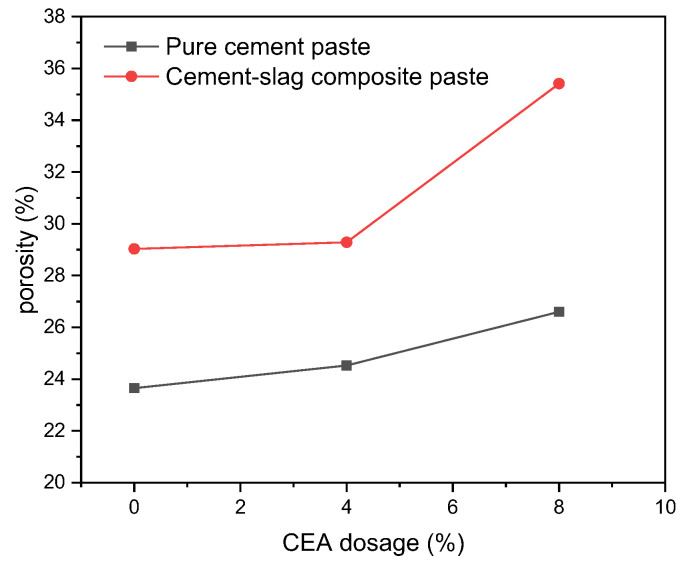
Effect of CEA on porosity of cement paste.

Figure 9 shows the porosities of pure cement paste and cement–slag composite paste with different CEA dosages at 90 d. With the increase of CEA dosage, the porosity of cement paste gradually increased. When the dosage of CEA was the same, the porosity of cement–slag composite paste was greater than that of pure cement paste. Therefore, the 90 d strength of cement paste decreased with the increase in CEA dosage. Moreover, when the CEA dosage was 4%, the 90 d strength of cement–slag composite paste was less than that of pure cement paste. It can be seen that although the hydration of cement and slag continues, the damage caused by the expansion stress in the early still exists after a long time of curing.

### 3.4. Hydration Products

**Figure 10:**
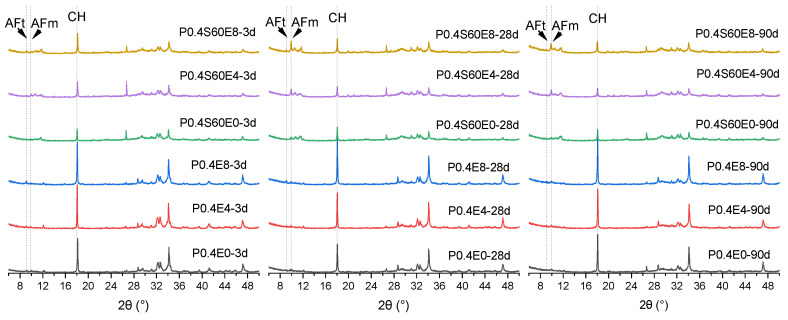
Effect of CEA on hydration products of cement paste.

Figure 10 shows the XRD patterns of cement paste with different CEA dosages at different ages. The peak intensity representing Ca(OH)_2_ (CH) and AFt (ettringite, 3CaO·Al_2_O_3_·3CaSO_4_·32H_2_O) of cement paste gradually increased with the increase of CEA dosage at 3 d. This means that the addition of CEA increases the content of Ca(OH)_2_ of cement paste, which is mainly due to the hydration of f-CaO in CEA, and the hydration of a small amount of calcium sulphoaluminate in CEA will also produce a small amount of AFt. With the increase of age, the peak intensity representing Ca(OH)_2_ and AFt of cement–slag composite paste gradually decreased, while the peak intensity representing AFm (monosulfoaluminate, 3CaO·Al_2_O_3_·CaSO_4_·xH_2_O) gradually increased. It may be that the pozzolanic reaction consumed part of Ca(OH)_2_, but from the result of the expansion rate of cement paste, the reduction of Ca(OH)_2_ content did not make the expansion shrink backward. In addition, the hydration of slag provided more aluminum phase and promoted the conversion from AFt to AFm. In the chloride environment, the increase of AFm is conducive to chemically binding more chloride ions, delaying the transmission of chloride ions, and improving the chloride ion resistance of cementitious materials.

**Figure 11:**
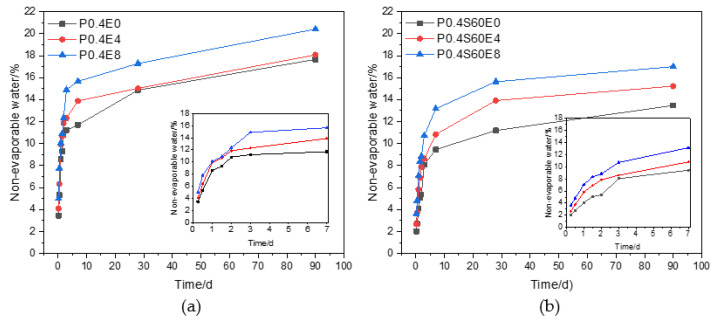
Effect of CEA on non-evaporable water content of cement paste: (**a**) pure cement paste; (**b**) cement–slag composite paste.

The content of non-evaporable water in cement paste is positively proportional to the degree of hydration to a certain extent [25]. Figure 11 plots the non-evaporable water content with the age of pure cement paste and cement–slag composite paste with different CEA dosages. The non-evaporable water content of cement paste increased rapidly within 3 d and then gradually increased with the increase of age. In addition, the non-evaporable water content of cement paste increased gradually with the increase of CEA dosage. This shows that cement paste rapidly hydrates within 3 d, and the addition of CEA can accelerate the hydration of cement paste and increase the hydration degree of cement paste.

## 4. Discussion

### 4.1. Influence of Expansion on Strength of Cement Paste

The comprehensive analysis of the expansion rate and strength of cement paste showed that when the CEA dosage was small, the expansion rate of cement paste gradually increased with age, and the early strength of cement paste slightly increased. When the dosage of CEA continued to increase, the expansion rate of cement paste increased sharply at the early age, and the early strength and late strength of cement paste decreased significantly. When the dosage of CEA was the same, the strength reduction of cement–slag composite paste was greater than that of pure cement paste. Therefore, the expansion of cement paste had a significant impact on the strength of cement paste, and the influence factors were mainly CEA dosage, cement paste type, and curing age.

**Figure 12:**
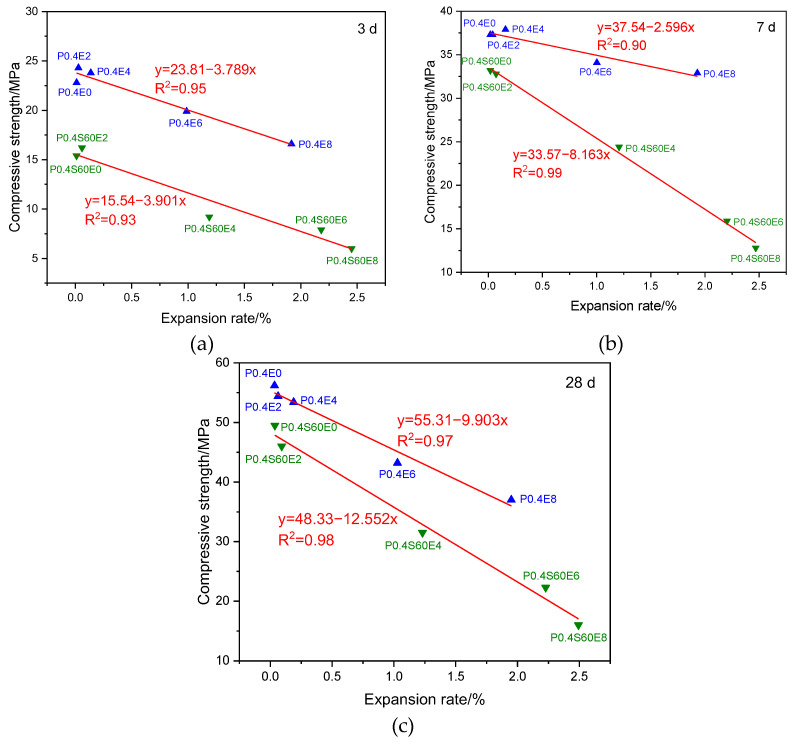
Effect of expansion on the strength of cement paste: (**a**) 3 d; (**b**) 7 d; (**c**) 28 d.

Figure 12 shows the relationship between the expansion rate and compressive strength of cement pastes with different CEA dosages at different ages. At different ages, whether pure cement paste or cement–slag composite paste, the expansion rates of cement pastes with different CEA dosages have a linear relationship with the compressive strengths, and the compressive strength gradually decreases with the increase of the expansion rate. When the expansion rate of cement paste is similar, the compressive strength of pure cement paste is greater than that of cement–slag composite paste. Theoretically, the larger the dosage of CEA, the greater the expansion stress and the greater the expansion rate of cement paste. When the expansion stress is greater than the tensile strength of cement paste around CEA, microcracks occur. As shown in Figure 2 and Figure 6, when the CEA dosage was large, the expansion rate of cement paste increased significantly, and microcracks occurred at the same time. The larger the CEA dosage, the greater the expansion rate and the more microcracks. Therefore, as shown in Figure 2 and Figure 4, the larger the CEA dosage, the greater the expansion rate and the smaller the compressive strength. With the increase of age, although the continuous hydration of cementitious materials can repair some microcracks, the compressive strength of cement paste still gradually decreases with the increase of CEA dosage.

### 4.2. Influence of Strength on Expansion of Cement Paste

When the dosage of CEA was 4%, the expansion rate of cement paste at the same curing age gradually increased with the increase of the content of slag and fly ash. At an early age, adding slag and fly ash will reduce the compressive strength of cementitious materials, and with the increase of slag and fly ash content, the compressive strength gradually decreases [26]. Therefore, the strength of cement paste had a significant impact on the expansion.

**Figure 13:**
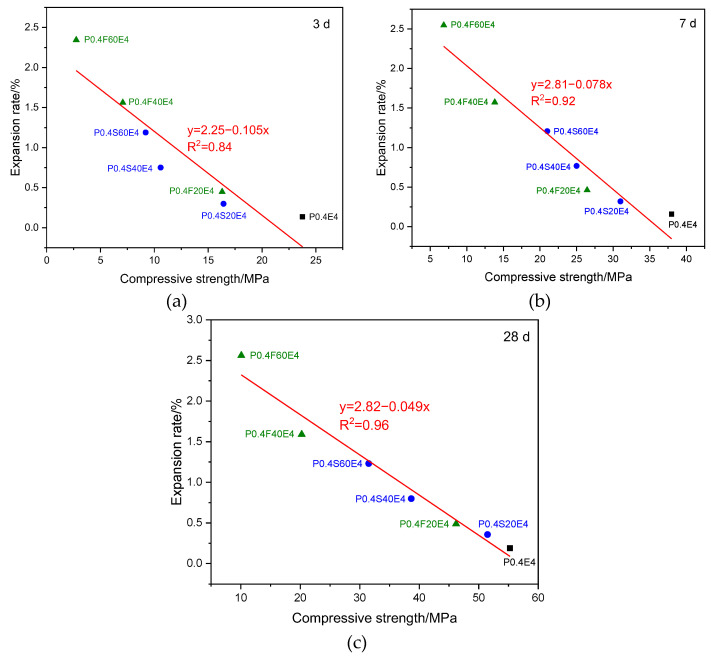
Effect of strength on expansion of cement paste: (**a**) 3 d; (**b**) 7 d; (**c**) 28 d.

Figure 13 shows the relationship between compressive strength and expansion rate of cement paste with the same CEA dosage at different ages. The compressive strengths and expansion rates of cement pastes are linear at the same age, and the expansion rate of cement paste decreases with the increase of compressive strength. With the increase of age, the linear relationship between compressive strength and expansion rate gradually enhances. Theoretically, when the dosage of CEA is the same, the expansion energy produced by hydration of CEA in cement paste is the same, and the strength of cement paste restricts the expansion. The smaller the strength, the greater the expansion rate of cement paste. The addition of slag and fly ash can reduce the early strength of cement paste. As shown in Figure 7, when the strength of the cement–slag composite paste was small, it was not enough to restrain the expansion stress, resulting in microcracks inside cement paste. The occurrence of microcracks further reduced the strength of cement paste. However, there were no significant microcracks inside the pure cement paste. These cause the data points representing cement paste with slag and fly ash in Figure 13a to shift to the left (indicating reduced compressive strengths) as a whole, far away from the data points representing the pure cement paste, making R^2^ smaller. With the increase of age, the strength of cement paste gradually increased, the constraint on expansion stress gradually increased, and the expansion rate slowly increased. In addition, significant pozzolanic reaction occurred in the late age of cement paste with slag and fly ash, and some microcracks were repaired, making the strength of cement paste with slag and fly ash gradually close to that of pure cement paste. Therefore, as shown in Figure 13b,c, the data points representing cement paste with slag and fly ash gradually shift to the right (indicating increased compressive strengths), close to the data points representing the pure cement paste, making R^2^ gradually increase.

## 5. Conclusions

(1) The expansion rate of cement paste increased with the increase of CEA dosage. The expansion rate of cement paste containing the same CEA dosage also increased gradually with the increase of slag and fly ash content.

(2) Adding low dosage of CEA slightly increased the early strength of cement paste, but the late strength of cement paste gradually decreased with the increase of CEA dosage. The strength of cement paste containing the same CEA dosage gradually decreased with the increase of slag and fly ash content.

(3) Adding low dosage of CEA slightly increased the early compactness of cement paste. With the increase of CEA dosage, the compactness of cement paste decreased sharply within 3 d and then increased gradually. The addition of CEA reduced the content of harmless pores in cement paste and increased the content of less harmful pores and harmful pores, and the porosity of cement paste gradually increased with the increase of CEA dosage. Adding CEA increased the hydration degree of cement paste.

(4) There was a linear relationship between the expansion rate and compressive strength of cement paste with CEA. The expansion of cement paste led to the generation of microcracks, and the compressive strength of cement paste decreased linearly with the increase of expansion rate. The strength of cement paste restricted the expansion, and the expansion rate of cement paste decreased linearly with the increase of compressive strength.

(5) When CEA is used to compensate shrinkage in cementitious materials, it is crucial to consider the impact of CEA on the strength of the cementitious materials, ensuring a harmonious development of expansion and strength. This will be a future research direction.

## Figures and Tables

**Table 1:** Chemical compositions of cement, slag, fly ash, and CEA (wt.%).

Oxide	Cement	Slag	Fly Ash	CEA
Al_2_O_3_	4.94	14.90	39.80	6.11
CaO	60.30	41.10	4.68	65.90
Fe_2_O_3_	3.38	0.83	6.15	1.89
K_2_O	0.41	0.49	0.50	0.56
MgO	2.10	6.74	0.51	2.04
Na_2_O	0.18	0.31	0.11	0.13
SO_3_	2.00	2.53	1.00	17.10
SiO_2_	23.13	31.60	42.20	5.19
TiO_2_	0.26	0.56	1.94	0.28
LOI	1.50	0.60	6.34	0.40

**Table 2:** Mix proportions of cement pastes.

No.	Cement/%	Slag/%	Fly Ash/%	CEA/%	w/b
P0.4E0	100	0	0	0	0.4
P0.4E2	98	0	0	2	0.4
P0.4E4	96	0	0	4	0.4
P0.4E6	94	0	0	6	0.4
P0.4E8	92	0	0	8	0.4
P0.4S60E0	40	60	0	0	0.4
P0.4S60E2	39.2	58.8	0	2	0.4
P0.4S60E4	38.4	57.6	0	4	0.4
P0.4S60E6	37.6	56.4	0	6	0.4
P0.4S60E8	36.8	55.2	0	8	0.4
P0.4S20E4	76.8	19.2	0	4	0.4
P0.4S40E4	57.6	38.4	0	4	0.4
P0.4F20E4	76.8	0	19.2	4	0.4
P0.4F40E4	57.6	0	38.4	4	0.4
P0.4F60E4	38.4	0	57.6	4	0.4

## Data Availability

The original contributions presented in the study are included in the article; further inquiries can be directed to the corresponding author.
